# Observations on the Prevalence, Characteristics, and Effects of Self-Treatment

**DOI:** 10.3389/fpubh.2016.00069

**Published:** 2016-04-18

**Authors:** Yinjun Zhao, Shuangge Ma

**Affiliations:** ^1^Department of Biostatistics, Yale University, New Haven, CT, USA

**Keywords:** self-treatment, prevalence, associated factors, treatment effects, empirical observations

## Abstract

**Aims:**

When facing illness, a person may choose self-treatment as an alternative to hospital (and primary care)-based treatment. Despite its important role in health care, the study on self-treatment remains limited. The goal is to collectively report the observations in the literature on the prevalence, characteristics, and effects of self-treatment.

**Methods:**

Databases (Medline/PubMed and Google Scholar) were searched. Articles were scrutinized for country of origin, sample size, recall period, prevalence, associated factors, etc.

**Results:**

Published studies have reported that in some regions, the prevalence of self-treatment is high and varies across illness conditions and treatment approaches. Self-medication is the most popular self-treatment approach. Multiple regional, demographic, personal, cultural, and religious factors have been implicated in the pursuit of self-treatment. In addition, accessibility of health care also plays a role. In general, self-treatment has a positive clinical and financial effect. However, there have been concerns on abuse and possible negative effects.

**Conclusion:**

This article reviews observations made in recent studies on several important aspects of self-treatment. Comprehensive and systematic study is still lacking. Interventions are needed to solve several problems associated with self-treatment.

## Background

When facing illness, a person may choose hospital (and primary care)-based treatment (which includes inpatient and outpatient treatments) or self-treatment. The definition of self-treatment differs in various publications. It equivalently means self-medication or self-care in some publications (1, 2), whereas it could be different from either of them (3, 4). According to the World Health Organization (WHO) (5), self-care is defined as “the activities that individuals, families, and communities undertake, with the intention of enhancing health, preventing illness, limiting illness, and restoring health.” By this definition, it is noted that a person pursuing self-care or self-medication may be for the purpose of enhancing health, and it is not necessary to have illness. In this article, self-treatment refers to “the scenario where a person uses non-prescription drugs or other approaches to cope with illness conditions” (3), which has the same meaning as “a treatment of oneself without professional help, to alleviate an illness or a condition” (4). By this definition, self-medication involves the utilization of drugs and is a special form of self-treatment.

Although hospital-based treatment offers a higher quality of care, self-treatment has the advantages of convenience and low cost. Unlike inpatient and outpatient treatments, data on self-treatment are usually not available in large medical databases. Self-treatment usually deals with minor illness conditions and has low cost per episode. In the literature, research on self-treatment is relatively limited. However, the role of self-treatment in health care should not be ignored, first because of its high prevalence, especially for certain common illness conditions. For example, a national survey conducted in 2008 in China reported that 27% of the respondents opted for self-treatment (2). In a survey conducted in Islamabad, Pakistan, 82% of the subjects chose self-treatment for headache (6). Second, although self-treatment has low cost per episode, it can result in considerable financial burden, because of its high frequency, and because the cost of self-treatment is usually poorly covered by insurance. For example, a recent study conducted in rural Beijing, China, reported that self-treatment expense accounted for 8% of the total per capita expense, almost as expensive as inpatient treatment (7). Third, studies have observed that, without the involvement of trained professionals, the effects of self-treatment have been mostly self-evaluated (8, 9), which may pose a public health concern.

In the literature, observations on self-treatment are scattered, and there has been a lack of collective and comprehensive discussions. To fill this knowledge gap, this study is conducted to selectively review and summarize observations made in recent studies on self-treatment. Of interest, the prevalence of self-treatment, factors that contribute to the decision of undertaking self-treatment, and clinical and financial effects were included. As the existing large databases with their special nature of data collection, usually contain information on inpatient and outpatient treatments, it is infeasible to conduct new data analysis, and the information needed has to be extracted from published studies.

## Methods

Compared to inpatient and outpatient treatments, literature on self-treatment is much limited. To identify relevant publications, we searched PubMed and Google Scholar using keywords including self-treatment, self-medication (which is the dominating form of self-treatment), and folk treatment for articles published between January 2010 and June 2015. The search covered title, keywords, abstract, and full text. The two authors did the selection lists separately and cross-check was used in every step of the search process in order to reduce the selection bias. The selection of eligible studies was completed in a two-stage process. The database search yield 1315 publications for review. Title examination showed that 1027 did not deal with self-treatment and were thus excluded. The remaining 288 articles underwent independent abstract review, and 220 were eliminated because they did not focus on prevalence, characteristics, or effects of self-treatment. A secondary search was conducted based on the first search results. Articles were scrutinized for country of origin, sample size, recall period, prevalence, associated factors, etc. Hand search of reference lists and citations of important articles resulted in an addition of 42 articles. The identified publication lists were then cross-checked by the two authors, and all the disagreements were solved. A total of 77 publications were finally selected based on these criteria. Since almost all the studies that we are interested are case reports or case studies based on surveys rather than evidence-based practices, and our goal is to provide a comprehensive discussion with multiple respects, we performed a literature review rather than a systematic review. Thus, all relevant articles were reviewed. With the limited number of available publications and the significantly different focuses of studies, our review is mostly descriptive as opposed to quantitative. As all information was extracted from published articles, no ethical approval was needed.

## Prevalence of Self-Treatment

There are multiple approaches of self-treatment. The most common is self-medication, which is the selection and use of medicines to treat self-recognized or self-diagnosed conditions or symptoms without physicians’ prescription (10). Folk treatment is another common approach, is prevalent in Asian countries, and includes acupuncture, aromatherapy, reflexology, massage, and others (11, 12). Different from other approaches, such as self-medication, folk treatment may involve other professionals. However, such professionals, especially in Asian countries, are usually not trained medical doctors. Thus, we also include folk treatment as a form of self-treatment. Spiritual or faith therapy and others have also been taken. Some of the existing studies investigate all self-treatment approaches as a whole, whereas others focus on a single approach or a single illness condition.

### Overall Prevalence

In some countries and regions, the overall prevalence of self-treatment is high, among which adolescent (13–17), elderly people (3, 18), and medical school students (19–26) are the special interesting populations. A national survey conducted in 2008 in China (2) suggested that the overall prevalence of self-treatment was 27%. In this study, self-treatment was defined as taking drugs and/or other home remedies or having a massage and/or physiotherapy, when experiencing symptoms during a period of 2 weeks prior to survey. In a survey study conducted in Miyun, a satellite city of Beijing, China, in 2012, out of 346 households, 75 had more than 5 episodes of self-treatments per person within 12 months (7, 12). In a survey conducted in Iran on weld workers (27), 90% stated a preference for self-treatment, 84.5% had used folk treatments, and 90% of the drugs used came from the stock of home pharmacies. Another population who drew our attention is pregnant women. In a study on abortion, 31.25% patients were found to have self-administered abortion pills (28). Besides, the prevalence that woman seek self-treatment when having menstruation problems was high, with only small proportions getting treatment from qualified physicians (29).

### Illness Condition-Specific Prevalence

Self-treatment is more common for some illness conditions, and condition-specific studies of self-treatment have also been conducted. A survey on the treatment of febrile illness in India showed that two-thirds of the patients either had folk treatments or purchased medicine from private pharmacies without prescription (30). A survey in Pakistan on the treatment of headache with 248 respondents showed that unprescribed pharmaceutical drugs (87.1%) were the most common treatment approach, whereas vitamins (3.4%), massage (4.5%), herbal remedies (2.2%), and homeopathic medicines (2.8%) were also adopted (6). Additionally, alcohol has been found to have pain-dampening effects, although the mechanism is unclear (31, 32). Other diseases with high self-medication prevalence include malaria (33–35), psoriasis (36, 37), oral disease (38), ophthalmology (39), and COPD (40).

### Approach-Specific Prevalence

There are also studies focused on specific self-treatment approaches. Self-medication, also known as over the counter (OTC), has been identified as the most important and most common approach (10, 16, 18, 41), and antibiotics are the most popular topics (15, 42–48). The most common non-prescription medications include pain killers, cough and cold medicines, anti-allergy medicines, and vitamins and energy tonics. A cross-sectional study conducted in South India (49) found that 71% of the subjects had used self-medication. In 6 months, the frequency of usage varied with a minimum of at least once to a maximum of five times and more. In a survey conducted in Hong Kong, 363 of 1102 (32.9%) respondents had purchased OTC over the past 3 months (1). The 2006 and 2007 Spanish National Health Survey reported that about 20% of all Spaniards indulge in self-treatment (50). Another study conducted in the urban areas of India (51) showed that the prevalence of self-medication in a period of 3 months was 11.9%. Fever (31%), headache (19%), and abdominal pain (16.7%) were the most common illness conditions for self-medication. In a study on suspected malaria patients in Kolasib, India (30), self-medicine from private pharmacies was taken by the majority of fever patients. Moreover, a couple of studies focused on the OTC consumption of women during their pregnancy, and the prevalence of self-medication ranged from 10.5 to 72.4% (52–54). A few studies take special interests in migrant workers or workers with vulnerable occupation, which shows that most of them adopted unsupervised self-medication in the treatment of their illness, whereas hospitals were usually the last resort for their health care (55–58).

Treatment using medical plants, also known as herbal remedies, has also been popular. In this review, we focus on the herbal medication that is not prescribed by physicians. A cross-sectional study in Turkey showed that the prevalence of herbal remedies was 39.2%, and lime, mint, rosehip, and lemon are the most commonly used herbs (59). A study in Northwest reported that although there was a decrease in the usage of some traditional medical plants, the decreasing process was not homogeneous (60). A study in a rural community in Nigeria found that 91.5% of children used local herbal remedies combined with medicine in the treatment of malaria (33). Herbal remedies were found to treat hypertension in South Africa (61).

Comparatively, folk treatments, such as massage and acupuncture, and other self-treatment approaches are less popular, and few studies have been conducted on its popularity. A cross-sectional survey conducted in Islamabad showed that compared to pharmaceutical drugs, there were still quite a lot of people who choose massage as their preference (9). In a study conducted on the middle-aged and elderly people in China, 9.2% subjects used folk treatment, such as scrapping and acupuncture (3).

## Factors Associated with Pursuing Self-Treatment

The prevalence of self-treatment varies significantly across population groups and regions. The decision making under the pursuit of certain health-care activities is a complex process. Multiple factors have been associated with the (variation in) prevalence of self-treatment.

### Regional Difference

Prevalence can vary across regions. It has been demonstrated that self-medication in rural areas is common (62), and a lot of studies have focused on rural areas (7, 33, 63). A national survey study conducted in China over a period of 4 years showed that prevalence was higher in urban areas than in rural China (2). Similar results were observed in a study conducted in India (49).

### Demographic and Personal Factors

Gender and race have been associated with the prevalence of self-treatment. A study conducted in Iran reported that females had a higher rate of using self-treatment than males (4). Similar results were reported in a study conducted in the coastal regions of India (49), rural area of Greek (63), Turkey (59), Spain (50), U.S. (64), and Iran (4). However, results in the literature have not been consistent. Opposite results were reported in the study conducted in urban India (51) and in a national study on analgesic use in Spain (50). Race difference was also found in the prevalence of self-treatment. In a study conducted in the U.S., Hispanics were found more likely to use complementary and alternative medicine (CAM) followed by blacks. Hispanics were more likely to use herbal medicines to self-treat cold and insomnia than Whites and Blacks and lower back pain than Whites (64).

Education and general knowledge contribute to the pursuit of self-treatment. A national self-treatment study in China (2) reported that people with higher education or with more medical knowledge tended to use self-treatment more frequently. Similar results were observed in a self-medication use study in Greek (63) and Iran (65) and in a herbal remedies use study in Turkey (59). Parents’ education was found positively associated with OTC use in adolescents (66). Opposite results were reported in a study on diabetes in Iran (4). No difference related to education level was found in the self-medication study on ophthalmology (67).

Self-treatment is more convenient and more affordable than hospital-based treatment. Various socioeconomic factors and health insurance status have been associated with self-treatment. In a study of topical ocular anesthetic abuse among Iranian welders (27), about 90% preferred self-treatment and quoted financial and cultural reasons. In a study on the middle-aged and elderly in China (68), the lack of time to see physicians was a vital reason for frequent self-medication among the young and literate. Cost and time constraints were the most common reasons for subjects to seek self-treatment (33, 38, 41, 49, 57, 65, 69). Other socioeconomic factors have also been associated with ­self-treatment. For example, several studies have identified income as a contributing factor (2, 4, 58, 70). A febrile illness study in India (30) reported that those with income higher than $104 per month had a higher self-treatment rate.

### Cultural and Religious Factors

Cultural and religious factors have been found to play an important role in some populations. The restriction that women belong to the overtly religious and feudal society contributed to the choice of self-treatment in females in Pakistan (6). Cultural norms that put an emphasis on outdated home-made remedies and faith healers as well as psychological dependence on painkillers and mood-altering drugs were also contributors (6). Self-treatment options were attractive to patients because they offered privacy, convenience, and autonomy (71).

### Accessibility and Others

Accessibility has been suggested as an associated factor. A study conducted in California, USA (72) suggested that the availability of health-care facility was associated with self-treatment. Similar results have been found in Ref. (33, 38, 41, 57, 65, 69). In addition, accessibility to unprescribed drugs was associated with the probability of self-treatment (2).

Other studies identified associated factors, including comorbid illnesses, perceived health status, attitude to illness and professional treatment, unhealthy lifestyles, physical activity involved in occupation, personal reference, and family members (4, 33, 41, 50, 59, 65, 69, 70). Varied illness symptoms, severity, and duration showed a negative association with the probability of self-treatment (2). In an Iran study on patients with diabetes mellitus, comorbid illnesses of hypertension, hyperlipidemia, and cardiac disease had a significant association with self-treatment (4).

## Effects of Self-Treatment

Multiple studies have demonstrated the positive medical effects of self-treatment (9, 16, 18, 39, 41). In a herbal remedies use study conducted in Hong Kong, almost 60% of subjects reported using a herbal remedy to overcome health problems or for health enhancement (59). Other self-treatment approaches have also been suggested as effective, such as massage, which was suggested to have good effects on the pain, walking ability, and quality of life of elderly people with knee osteoarthritis (8). Massage was also found to reduce muscle soreness combined with the use of a roller device (73).

Self-treatment can also reduce pressure on health-care services. For patients living in rural or remote areas and having limited access to health facilities, illness conditions can be controlled to a great extent with self-treatment (74). In the last 5 years, very few studies analyzed the role of self-treatment in shifting responsibilities and health-care cost from government and patients.

Several risks are potentially involved in self-treatment (10), including delays in seeking professional advice, incorrect self-diagnosis, infrequent but severe adverse reactions, dangerous drug interactions, incorrect manner of administration, incorrect dosage, incorrect choice of therapy, masking of a severe condition, and risk of abuse. A study in India reported that 93.5% of the surveyed subjects were not aware of potential side effects (51). Medical plants used in self-treatment may contain powerful active substances that can contribute to adverse events, including interactions with synthetic medicines (75). In a study conducted in Michigan, about one-third of the subjects were taking more than one medication and/or herbal product or supplement, which could increase their risk of drug–herb interactions (76). This was further complicated by the fact that 20% of the subjects did not inform their physicians of their self-treatment choices. A study on the adverse effect of acupuncture in China showed that 3.76% subjects had at least one adverse event during the treatment period. Older people and male had higher risk of having adverse event (77). There have been a lot of public and professional concerns on the abuse of self-treatment, especially in developing countries.

## Discussion

Based on the review results, prevalence of self-treatment, factors associated with pursuing self-treatment, and effects of self-treatment have been discussed. Despite the significant and practical importance of self-treatment, research on self-treatment is still very limited. Especially, there is a lack of collective and comprehensive discussion. In this article, we have attempted to collect the most relevant publications from PubMed and Google Scholar, two of the most major sources of biomedical publications. Our literature search suggested that although a large number of publications contain the word “self-treatment,” the dominating majority of them only mention self-treatment as an option for treating illness, and there is a lack of detailed discussion. As can be partly seen above, the research focuses of the reviewed studies differ significantly. Thus, this review does not follow the “classic” meta-analysis and systematic review. Rather, as pointed out by the reviewers, it is “less systematic” but has a more descriptive and opinion nature. On the other hand, it is no doubt that some descriptions shed few lights on adding our knowledge due to the “descriptive” and “big picture” characteristic. However, such a big picture provides us a panoramic view of multiple aspects of self-treatment, and it helps us to get a comprehensive understanding of the recent study domains. Moreover, this review contributes to find some potential problems or interesting topics indicated in current studies, which are worthy to explore in the future. Nevertheless, with the scarcity of comprehensive discussion on self-treatment, this study can still provide insightful information and complement the existing literature.

### Prevalence

With its unique nature, the prevalence of self-treatment is not as well measured and documented as inpatient and outpatient treatments. Survey studies suggest that the overall prevalence of self-treatment is high in some countries and regions. As indicated in the literature (2, 6, 27, 30), self-treatment can be much more common than inpatient and outpatient treatments among certain populations. With a lack of comprehensive study and very limited data for many countries, it is impossible to draw conclusion on the global self-treatment prevalence.

The scope of self-treatment is limited, and its prevalence varies significantly across illness conditions. There are a few studies focusing on the prevalence for certain minor to mild illness. Such studies are of interest to clinical practitioners and public health researchers. Self-treatment has been applied to various illness conditions, and only a few have been investigated in details. Research on other illness conditions can be informative and may be pursued in the future. It should be noted that the observed self-treatment prevalence for an illness condition may be only applicable to the studied population and region.

Self-medication has been identified as the most common approach. It is usually applied to minor or existing conditions. When a patient encounters a minor illness or has extensive knowledge of the illness, self-medication using OTC medicine can be effective. The study on existing conditions can be complicated. Patients may encounter the same conditions and purchase the same drugs without a new prescription but use the same prescription from the previous times or can purchase different drugs based on their own judgment. However, the two situations are different and have not been separately investigated in the existing studies. Medical plants and folk treatment have also been adopted, but less frequently. Spiritual therapy, wait and active watch, and a few other approaches have also been adopted. But the corresponding research has been very limited.

### Factors Contributing to Self-Treatment

Multiple factors have been associated with pursuing self-treatment. The observed regional differences can be explained by the difference in the availability of OTC medicine and other treatment approaches as well as confounded by demographic, socioeconomic, and cultural factors. Studies have suggested that demographic and personal factors are associated with health-care decision making, and this observation goes beyond self-treatment. As self-treatment has less professional involvement, personal characteristics can have even more important implications. The observed associations for gender, race, education, and knowledge level have been well discussed in the literature.

Certain unprivileged populations are more likely to have self-treatment. They have lower economic status, long and inflexible working hours, poor knowledge of medicine, limited or no health insurance coverage (or limited knowledge of the insurance system), or limited access to health-care facilities. The mechanism of pursuing self-treatment for these population is different from others. For the reason of lacking access to health facilities or lacking powerful health insurance coverage, self-treatment might be their first choice when facing illness. However, lower education or the lack of medicine knowledge might lead ineffective or harmful treatment, which worse their illness conditions and increase their economic burden. Policy interventions, for example, tuning of the health insurance system, are needed to improve access to professional health care for those people. Possible relationship between the health inequality and the pursuit of self-treatment is worthy to explore in the future.

In certain countries, cultural and religious factors play an important role. This is often observed in developing countries, making the study further complicated with possible confounding by the limited accessibility of health care. Compared with other factors, in the short run, these factors are not easily modifiable. Studies, especially quantitative studies, on the impact of such factors are still limited.

Different illness conditions may intervene with each other. If a condition makes a patient less mobile, he or she can be more likely to cope with other illness conditions using self-treatment. There are a few hospital-based large databases that contain information on multiple illness conditions. They can be potentially used to study the effect of one condition on the self-treatment of other illness conditions.

### Effects of Self-Treatment

The effectiveness of a treatment approach needs to be evaluated in multiple aspects, including medical, financial, social, and others. Several studies have suggested the medical effectiveness of self-treatment. Such an observation is reasonable, as self-treatment is mostly associated with minor illness conditions. However, the existing evaluations of medical effects have been mostly based on self-evaluation, and more rigorous evaluations by health-care professionals are needed. As it has a low cost per episode, self-treatment can have a positive financial effect. On the other hand, the cost of self-treatment is often not covered by health insurance. With a high frequency, the accumulated out-of-pocket cost may still be high. This may pose a problem, especially to the financially less advantaged. Policy interventions are needed to protect the (potentially detrimental) financial consequences of self-treatment. Without the supervision of professionals, self-treatment can be abused, which may have adverse medical and financial consequences. Our literature review suggests that studies, especially quantitative studies, on this subject are still limited.

### Limitations

This article covers multiple aspects of self-treatment, and the discussion on a single aspect may not be as extensive as in some more focused studies. Self-treatment is much more difficult to study than inpatient and outpatient treatments. For hospital-based treatments, there are large databases with extensive data collection. For self-treatment, all of the reviewed studies are based on surveys. Survey studies often have limitations, including limited sample sizes, potential bias in sample selection, inaccurate and limited information collection, and others. Self-treatment demonstrates significant variations across regions, populations, and illness conditions. Patients in different countries or different regions of the same country may apply different medicines to treat the same condition. In this sense, self-medication in one country may not be directly comparable to that in another. Because of this reason and the limited number of available studies, we have not attempted to conduct quantitative analysis, and for many reviewed studies, we have focused on the narrative observations. Although every effort has been made to avoid publication selection bias, there is still a risk. This is further complicated by the limited and unsystematic existing studies. Another limitation is that this literature review may not be as “evidence-based” as systematic review, and it is limited in providing a methodological reference as systematic review. However, we have tried every effort to encompass as more articles as possible and excluded and included them as objectively as possible. This review can provide a “big picture” and cover multiple aspects in which systematic review is hard to achieve. The investigation of self-treatment is still ongoing. Quite a few reviewed studies were conducted in the most recent years. This review may need to be updated in the near future.

## Conclusion

Self-treatment plays an important role in health care. However, research on self-treatment is very limited. In our literature review, it is found that self-treatment has a high prevalence, which varies across illness conditions and treatment approaches. Multiple factors are associated with pursuing self-treatment. Some of these factors are unique to self-treatment and deserve further attention. There have been studies evaluating the medical, financial, and social effects of self-treatment. More comprehensive and more systematic studies are needed, and policy interventions are needed to solve problems encountered in self-treatment. Despite a few limitations, this study, as one of the few reviews on self-treatment, can be insightful to public health and medical practitioners and policy makers, especially in a global health prospective.

## Author Contributions

Both authors are involved in study design and literature review and approved the final draft.

## Conflict of Interest Statement

The authors declare that the research was conducted in the absence of any commercial or financial relationships that could be construed as a potential conflict of interest.
